# Surveillance of antiviral resistance markers in Argentina: detection of E119V neuraminidase mutation in a post-treatment immunocompromised patient

**DOI:** 10.1590/0074-02760160262

**Published:** 2016-11-16

**Authors:** Andrea Pontoriero, Martín Avaro, Estefania Benedetti, Mara Russo, Andrea Czech, Natalia Periolo, Ana Campos, Ana Zamora, Elsa Baumeister

**Affiliations:** 1 National Influenza Centre PAHO/WHO, National Reference Laboratory, Respiratory Viral Diseases Service, Department of Virology, National Institute of Infectious Diseases, National Agency of Laboratories and Institutes of Health Dr Carlos G Malbrán, Ciudad de Buenos Aires, Buenos Aires, Argentina; 2 University of Tucuman, School of Pharmacy and Biochemistry, Microbiology Institute, Tucuman, Argentina

**Keywords:** influenza resistance, Argentina, E119V substitution, surveillance

## Abstract

Although vaccines are the best means of protection against influenza, neuraminidase inhibitors are currently the main antiviral treatment available to control severe influenza cases. One of the most frequent substitutions in the neuraminidase (NA) protein of influenza A(H3N2) viruses during or soon after oseltamivir administration is E119V mutation. We describe the emergence of a mixed viral population with the E119E/V mutation in the NA protein sequence in a post-treatment influenza sample collected from an immunocompromised patient in Argentina. This substitution was identified by a real-time reverse transcriptase polymerase chain reaction (RT-PCR) protocol and was confirmed by direct Sanger sequencing of the original sample. In 2014, out of 1140 influenza samples received at the National Influenza Centre, 888 samples (78%) were A(H3N2) strains, 244 (21.3%) were type B strains, and 8 (0.7%) were A(H1N1)pdm09 strains. Out of 888 A(H3N2) samples, 842 were tested for the E119V substitution by quantitative RT-PCR: 841 A(H3N2) samples had the wild-type E119 genotype and in one sample, a mixture of viral E119/ V119 subpopulations was detected. Influenza virus surveillance and antiviral resistance studies can lead to better decisions in health policies and help in medical treatment planning, especially for severe cases and immunocompromised patients.

Influenza virus is a major human pathogen associated with high morbidity and mortality both in temperate and subtropical/tropical regions. Since the outbreak of the 2009 influenza pandemic, the A(H3N2) and A(H1N1)pdm09 subtypes have become the two major influenza virus strains, along with influenza B viruses that cause the disease in humans. The emergence of A(H1N1)pdm09 prompts a better evaluation of the genetic characteristics of influenza viruses and improvement of the surveillance of circulating influenza viruses resistance to existing antiviral drugs. The A(H3N2) subtype has circulated and caused influenza in human populations before the emergence of A(H1N1)pdm09 subtype. Antiviral drugs constitute an important tool to control these infections. The neuraminidase inhibitors (NAIs) oseltamivir and zanamivir have been the cornerstone of anti-influenza therapy in recent years. However, their effectiveness has been compromised by rapid emergence of resistance among some A(H3N2) and A(H1N1) viruses circulating in different geographic regions (Okomo-Adhiambo et al. 2010). One of the most frequent changes in A(H3N2) viruses during or soon after oseltamivir administration has been reported to be the amino acid substitution of glutamic acid (E) to valine (V) at position 119 of neuraminidase (NA) (Eshaghi et al. 2014). A recent study, based on the experiments on viruses collected via the Global Influenza Surveillance and Response System (GISRS) and analysed by five World Health Organization Collaborating Centres (WHO CCs) between May 2014 and May 2015, demonstrated that from a total of 13,312 viruses examined, approximately 0.5% (n = 68) showed highly reduced inhibition by at least one of the four NAIs tested and another 56 isolates exhibited moderately reduced inhibition (Hurt et al. 2016).

An immunocompromised host is a patient who does not have the ability to respond normally to an infection due to weakened immune system. This inability to fight infection makes these patients susceptible to bacterial, fungal, and viral infections, such as influenza (Eshaghi et al. 2014). Antiviral prophylaxis and therapy are particularly important in these patients because the influenza vaccine is often poorly immunogenic and unlikely to be fully protective (Yousuf et al. 1997). In addition, these patients are at risk of developing antiviral resistance and subsequent complications. It is worth pointing out that although there is a stable low rate of oseltamivir and zanamivir resistance among circulating influenza A viruses, a higher prevalence of drug-resistant influenza viruses in severely immunosuppressed or immunocompromised patients undergoing antiviral treatment has been well documented (Abed et al. 2009, Memoli et al. 2010). A(H3N2) viruses showing oseltamivir resistance due to the E119V NA mutation have previously been detected in immunocompromised paediatric patients undergoing oseltamivir treatment. Antiviral therapy in immunocompromised patients is often associated with prolonged viral shedding (Ruiz-Carrascoso et al. 2010). In some cases, the E119V oseltamivir-resistant variants were selected within a few days of oseltamivir treatment, whereas in other cases, many weeks of cumulative treatment were undertaken before the E119V variant was detected (Hurt et al. 2013). Mixed viral populations that contained sensitive and resistant viruses in different proportions have also been detected in intra-treatment samples collected from paediatric patients (Valinotto et al. 2010).

In this report, we describe the emergence of the E119V substitution in the NA protein of an influenza A(H3N2) isolate detected in a clinical specimen collected from an immunocompromised patient after oseltamivir treatment in Argentina during 2014 epidemic season.

## MATERIALS AND METHODS

*Patients and data collection* - Weekly influenza surveillance is routinely conducted by the Respiratory Viruses Laboratory at the National Institute of Infectious Diseases, National Agency of Laboratories, and Institutes of Health (ANLIS) Dr Carlos G Malbrán, a WHO National Influenza Centre (NIC), through its National Influenza and Respiratory Virus Surveillance Network (NIRN). This Network, which comprises 65 laboratories distributed all over the country, usually processes respiratory samples such as swabs and nasopharyngeal aspirates (NPA), and examines them by immunofluorescence methods using commercial kits for detection of the respiratory syncytial virus, adenovirus, parainfluenza virus 1-3, influenza A and B viruses. Besides, some laboratories (n = 35) carry out influenza virus detection by quantitative reverse transcriptase polymerase chain reaction (qRT-PCR) using specific primers and probes recommended by Centres for Disease Control and Prevention (CDC). All influenza A and B clinical specimens detected by the NIRN are routinely submitted to the NIC located in Buenos Aires for isolation and further antigenic and genomic characterisation. All the results obtained are compiled by the Ministry of Health in the national database called SIVILA (National System of Laboratory Surveillance). Clinical data [regarding influenza-like illness (ILI) and pneumonia] are also collected by the National System of Health Surveillance. These data come from clinical notifications that public and private hospitals communicate to the Ministry of Health.

*Viral RNA and subtyping* - Extraction of total nucleic acid from clinical specimens was performed at the NIC using the QIAamp® Viral RNA Mini Kit (QIAGEN), according to the manufacturer’s instructions. Influenza A virus subtypes H3 and H1pdm09 were identified by qRT-PCR using a SuperScript III Platinum One-Step qRT-PCR system (Invitrogen), specific primers, and FAM-BHQ1 dual-labelled probes developed by CDC (Shu et al. 2011).

*Molecular antiviral assays* - In 2014, a rapid genotypic screening was implemented at the NIC to identify the single nucleotide polymorphism (SNP) encoding E119V substitution in clinical specimens that contained A(H3N2) influenza virus. This technique allows the differentiation between wild-type (WT) and oseltamivir-resistant viruses. The original protocol was provided by Dr Adam Meijer, National Institute for Public Health and the Environment, The Netherlands. It was essentially a qRT-PCR assay conducted using a reaction mixture that included specific primers for influenza A(H3N2) NA segment with the E119V coding region and a pair of dual-labelled probes for the detection of the E119 and V119 variants.

*Sanger sequencing and phylogenetic analysis* - The NA segment (1-1052 bp) of the viruses from pre- and post-treatment samples was sequenced to confirm the results obtained by E119V SNP screening. The haemagglutinin (HA) segment of the E119V variant was also sequenced (1-986 bp). The sequencing PCR amplicons were purified with the MinElute® Gel Extraction Kit (QIAGEN). Sequencing reactions were performed using the BigDye® Terminator v3.1 Cycle Sequencing Kit (Applied Biosystems™), and the products were analysed on an ABI PRISM® 3700 Genetic Analyser (Applied Biosystems™).

Sequencing reaction conditions and primer sequences are available on request.

Sequences were analysed using BioEdit software, version 7.0.5.3 (http://www.mbio.ncsu.edu/BioEdit/bioedit.html). MEGA 4 (Tamura et al. 2007) and MEGA 5 (Tamura et al. 2011) programmes were used to build the phylogenetic trees using the neighbour-joining distance method. Tree topology was supported by bootstrap analysis with 1000 replicates. Vaccine strain sequences, as well as sequences from viruses collected in other countries, were obtained from the EpiFlu database available via the Global Initiative on Sharing All Influenza Data (GISAID) website, https://www.gisaid.org, and included in the analysis.

*Ethics* - Written informed consent and explicit ethical approval were not sought because this was an observational study undertaken as part of the routine virological surveillance (anonymously, without identification of the patients). This procedure is indicated in the Terms of Reference for WHO NICs, which describe the basis of the WHO surveillance in more than 130 countries (Pontoriero et al. 2011).

## RESULTS

**Fig. 1:**
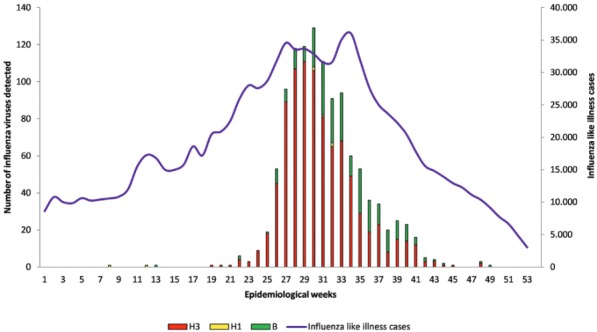
: total number of influenza viruses and influenza-like illness cases detected in Argentina in 2014. Data are presented by epidemiological week.

In 2014, the NIRN analysed a total of 51,744 samples for the detection of respiratory virus isolates that came from paediatric and adult inpatients and outpatients with acute lower and upper respiratory tract infections. The samples were accompanied by the clinic-epidemiological forms containing at least some basic information, such as patient initials, gender, age, city/state of onset of illness and the dates of the initiation of the symptoms and sample collection. Of these, 1,679 (3.24%) samples tested positive for influenza virus. This year, from these samples, the NIC received a total of 1,140 influenza virus isolates positive clinical specimens. The mean age of donors was 43 years (range: one month to 92 years); 54% of donors were men. Out of these influenza samples received at the NIC, 888 (78%) contained A(H3N2) virus, 244 (21.3%) had influenza type B viruses, and 8 (0.7%) were positive for A(H1N1)pdm09. In 2014, influenza viruses circulated all year round in Argentina, and their main activity was observed from June to December. The total number of influenza viruses detected and ILI cases registered, by epidemiological week (EW) in 2014, is detailed in Fig. 1. The A(H3N2) virus was first detected in May and progressively became the most frequent influenza A subtype circulating in Argentina. A(H3N2) activity was registered in the period from June to September 2014 (EW 23-EW 37), with a peak in EW 29 and subsequent decrease in frequency to a very low level until the end of the year. Influenza A(H1N1)pdm09 virus co-circulated at a frequency much lower than that of the A(H3N2) and influenza B viruses. The first peak of ILI cases registered in EW 27 perfectly matched the surge in A(H3N2) activity. Meanwhile, the second peak in EW 34 was related to the co-circulation of influenza A and B viruses.

**Fig. 2:**
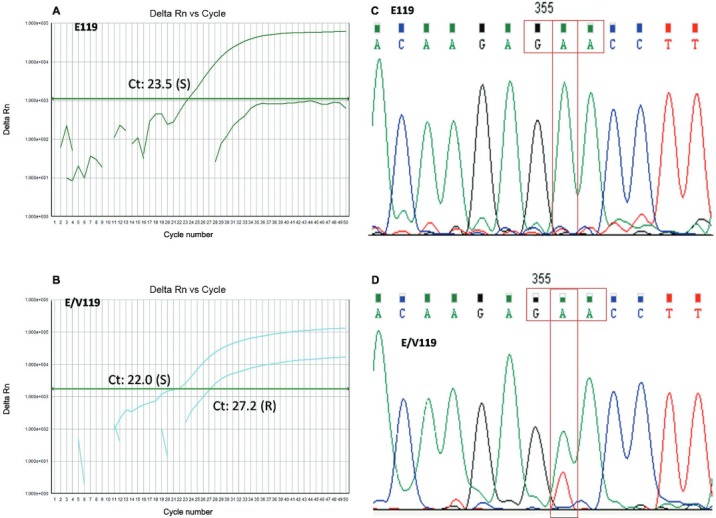
: (A) a quantitative reverse transcriptase polymerase chain reaction (qRT-PCR) amplification plot corresponding to the wild-type E119 virus; (B) a qRT-PCR amplification plot corresponding to the mixture of E119 and V119 viral subpopulations; (C) a Sanger sequencing chromatogram showing base substitutions in the codon for residue 119 of the neuraminidase gene in the pre-treatment sample; and (D) Sanger sequencing chromatogram of the post-treatment sample; sensitive/resistant E119E/V subpopulations.

Out of 888 A(H3N2) samples received at the NIC, 842 were tested for the E119V substitution: 836 samples were collected from untreated patients and six samples were obtained from six patients (four of them immunocompromised) after treatment. WT E119 genotype was found in 841 specimens and one sample contained a mixture of viral E119/V119 subpopulations. This sample came from a post-treatment specimen collected from an immunocompromised patient. The sample was sent to the NIC by an NIRN laboratory member located in Tucuman, a province in the north of the country. Data collected showed that this specimen was from a 12-year-old girl with a history of acute B-cell lymphoblastic leukaemia. On September 15th, the first NPA tested positive for influenza A virus by qRT-PCR analysis carried out in Tucuman, and oseltamivir therapy (75 mg twice a day orally) was administered from that date until September 20th. On September 25th, the second NPA of the same patient also tested positive for influenza A virus. First and second samples were sent to the NIC. Unfortunately, none of these viruses could be recovered in tissue culture and phenotypic studies could not be performed. Sanger sequencing of both pre- and post-treatment samples confirmed the results obtained by E119V SNP screening and further quantitative assays will be necessary to confirm this finding (Fig. 2). Nucleotide sequences of all the genes sequenced have been deposited in the GISAID database under accession numbers EPI_ISL_172694 and EPI_ISL_172636. The HA sequence analysis of the resistant virus demonstrated that this strain belongs to group 3C.3 and is different from the A(H3N2) strain included in the 2014 influenza vaccine (McCauley et al. 2015).

## DISCUSSION

Although the frequency of the E119V substitution is low (0.01%) (WHO 2015), in this study, we have been able to detect a mixture of E119/V119 subpopulations in an influenza A(H3N2) virus isolate recovered from a post-treatment immunocompromised patient. This variant had been observed previously by other researchers (Hurt et al. 2013, Eshaghi et al. 2014). Because the resistant variant was not recovered in tissue culture, phenotypic assays could not be performed. Molecular detection of the E119V SNP cannot alone inform about oseltamivir resistance without complementary studies to confirm the decrease of the susceptibility to antiviral drugs. Based on published data, the clinical impact of this polymorphism is not clear, but it may depend on the proportion of the drug-resistant (V119) variant (WHO 2015). However, following the recommendation of the WHO working group on the surveillance of influenza antiviral susceptibility, any detection of V119 variant is critical to the evolution of the patients and should result in a prompt treatment review (WHO 2015).

The absence of the E119V change in the pre-treatment sample suggests that the V119 variant arose as a consequence of the oseltamivir therapy. This finding is in agreement with previous reports about the emergence of E119V substitution in oseltamivir-treated immunocompromised patients infected with influenza A(H3N2) virus (Ison et al. 2006). The risk of developing resistance during antiviral treatment is considerably higher in the immunocompromised patients than in immunocompetent ones (Hurt et al. 2012). Because of this, samples for NAIs testing should be taken specially from the immunocompromised patient undergoing influenza treatment. These samples should be analysed immediately using molecular-based techniques for common resistance-associated mutations [e.g., E119V in A(H3N2) and H275Y in A(H1N1)pdm09 viruses], and prompt feedback must be given to treating clinicians, so that alternative therapies can be initiated (Hurt et al. 2013). The implementation of a rapid molecular technique for the detection of known substitutions, that confer antiviral resistance using reagents and equipment available at the NIC, represents a valuable tool for antiviral susceptibility monitoring, especially for testing pre- and post-treatment influenza positive clinical specimens.

This first study represents the beginning of the National systematic surveillance of the antiviral susceptibility of circulating influenza A(H3N2) viruses in Argentina that will contribute to better decisions in health policies and help in selecting optimal medical treatment. Within the frame of the WHO Global Influenza Surveillance, the NICs have the responsibility for early detection of variants associated with antiviral resistance in individual patients, and cluster populations that may have implications on public health. The national data generated and analysed in this study may help improve the knowledge about the resistance of influenza viruses to antiviral drugs, which is a global concern.
